# Electrocardiographic Changes, Mortality, and Late Period Findings in Methyl Alcohol Poisoning

**DOI:** 10.3390/jcm13195999

**Published:** 2024-10-08

**Authors:** Abuzer Coskun, Burak Demirci, Ismail Oymak, Enes Ferhatlar, Sevki Hakan Eren

**Affiliations:** 1 Department of Emergency Medicine, Istanbul Bagcilar Education and Research Hospital, Istanbul 34200, Turkey; drburakdemirci@hotmail.com (B.D.); drismailoymak@gmail.com (I.O.); enesferhatlar@gmail.com (E.F.); 2 Department of Emergency Medicine, Faculty of Medicine, Gaziantep University, Gaziantep 27310, Turkey; shakaneren@gmail.com

**Keywords:** emergency department, methyl alcohol poisoning, QTc, late findings

## Abstract

**Background**: Methyl alcohol poisoning (MAP) is a common commercial compound that can lead to significant morbidity and mortality when exposed to high levels. This study aims to describe MAP-related electrocardiography (ECG) changes and post-acute late complications. **Materials and Methods**: The study was conducted through a retrospective data review between 2017 and 2023. Patient data were recorded, including demographic information, medication use, and laboratory results. Twelve-lead ECG recordings were evaluated and the results were recorded. The cases included in the study were grouped according to QTc distance, ECG findings, late-term complications, treatment status, and mortality. **Results**: The mean age of all cases included in the study (n = 227) was 43.23 ± 11.11 years, 8 (3.5%) cases were female, and the age distribution was between 19 and 68 years (*p* = 0.792). The age distribution of QTc groups was not significant (*p* = 0.792). The mean QTc distance was 442.7 ± 60.1 ms in all cases (n = 227) and 514.08 ± 5.45 ms in cases with mortality (n = 49) (*p* < 0.001). The mean time to application of the patients to the hospital (n = 227) was 19.1 ± 4.61 h, and blood sugar was 130.7 ± 32.09 mg/dL (*p* < 0.001). In addition, the mean pH of the cases (n = 227) was 7.14 ± 0.2, bicarbonate was 17.17 ± 4.86 mmol/L, the base deficit was −6.21 ± 3.18 mmol/L, the anion gap was 19.36 ± 7.31 mmol/L, and lactate was 4.82 ± 2.45 mmol/L (*p* < 0.001). Mortality occurred in 49 (21.6%) of the patients, and all of them were in-hospital deaths. In all cases where mortality occurred, pH was below 6.93 ± 0.22 and severe acidosis was directly related to death. MAP, sinus tachycardia in 31 (13.7%) cases, bradycardia in 8 (3.5%), atrial fibrillation in 5 (2.2%), accelerated idioventricular rhythm in 3 (1.3%), and 11 (4.8%), left bundle branch block, and right bundle branch block were detected in 22 (9.7%). All of these ECG findings were newly developed conditions with no previous history. In the 6-month follow-up after discharge, 4 (1.8%) of the cases developed neurological deficit, 15 (6.6%) had acute coronary syndrome and severe heart failure, 23 (10.1%) had permanent blindness, 6 (2.7%) had renal failure, and 6 (2.7%) had pancreatitis. **Conclusions**: Methyl alcohol poisoning can cause various ECG changes; sinus tachycardia, nonspecific changes, and QTc prolongation are the most common findings. These changes are more pronounced in cases of severe acidosis. Patients should be warned of late signs of MAP.

## 1. Introduction

In general, the most common alcohol poisonings are ethanol, isopropyl alcohol, methyl alcohol, and ethylene glycol. While ethanol and isopropyl alcohol are the primary toxic components, methyl alcohol and ethylene glycol are the metabolites that cause toxic effects, have a more severe clinical course, and have a high risk of morbidity and mortality. Although their diagnosis, follow-up, and treatments are similar, emergency room visits may occur with different clinical presentations depending on the amount of exposure, duration, and characteristics of the person [1]. Particularly in recent years, illicit alcohol production has utilized methyl alcohol (MA), a toxic form of alcohol. Consumption of MA can lead to severe poisoning and death. Charcoal distillation produces MA [2]. Because of its solvent effect, it is widely used in the industrial sector, particularly in dry cleaning, automotive, fuel, antifreeze, glass cleaner, and paint thinner. Consequently, it is permissible to sell. It is difficult to differentiate from ethyl alcohol when consumed orally, as it is colorless and odorless. It is particularly suitable for the production of alcoholic beverages due to its lower cost than ethyl alcohol. The frequency of emergency service admissions for methyl alcohol poisoning (MAP), caused by illicit alcohol consumption, has increased in recent years [3].

MAP is a substantial cause of morbidity and mortality [4]. Because methyl alcohol is easy and cheap to produce, oral poisoning from bootleg alcohol is common. In rare cases, industrial production can lead to accidental oral ingestion. It has also been reported that it can cause poisoning by inhalation or dermal route [3,4]. The enzyme alcohol dehydrogenase converts MA to formaldehyde. The aldehyde dehydrogenase enzyme [5] catalyzes the conversion of formaldehyde to formic acid. Formic acid has a half-life of approximately 30 h [6]. In humans, the lethal dose of MA ranges from 15 to 500 mL [7]. In addition, the MAP mechanism encompasses the expression of proteins, the production of pro-inflammatory cytokines, cellular homeostasis, lipid peroxidation, and oxidative stress [8,9,10]. Central nervous system depression, headache, vertigo, nausea, vomiting, and lack of coordination commence following the consumption of MA. The second set of symptoms, which includes acidosis and blurring/loss of vision, commences within the 10th to 30th hour of exposure after the initial symptoms [11]. Untreated MAP damages the optic nerve and retinal epithelial cells, resulting in blurred vision or, in severe poisoning, blindness. Characteristic symptoms include central scotoma, redness, optic disc pallor, and papilledema [3]. Formic acid accumulation causes these symptoms, potentially leading to respiratory failure and death [7]. The diagnosis of MAP is based on clinical signs and symptoms, acid-base status, direct serum methyl alcohol levels, anion, and osmolar deficit. Treatment for MAP often involves the use of ethanol, fomepizole, sodium bicarbonate, and hemodialysis [12,13].

Although methyl alcohol poisoning has long been known, little is known about its effects on the cardiovascular system and electrocardiogram (ECG) findings [4]. The underlying causes of MAP, cardiovascular, and ECG changes may be metabolic acidosis and formic acid accumulation [6]. A 2022 study suggests that cardiac causes related to MAP could include severe acidosis, decreased fibrinogen level, disseminated intravascular coagulation, decreased extracellular pH, and endothelial dysfunction [14]. When someone is severely drunk, their ECG often shows problems such as atrioventricular block, PR interval prolongation, and QT interval prolongation, which can often lead to torsades de pointes. In addition, sinus tachycardia is a frequently observed phenomenon [15]. In a case presented by Dibajnia et al. [16], ECG findings at the time of initial evaluation were indicative of myocardial infarction, as the patients exhibited newly found ST segment elevation and T wave inversion. One of the few literatures is Jaff et al. [4], who reported an ECG study conducted on nine patients with MAP in 2014. The other is Sanaei-Zade et al. [13], who presented their analysis of ECG findings in 42 patients with MAP in 2013.

This investigation, one of the larger studies, discloses MAP-related ECG findings. Furthermore, to the best of our knowledge, the literature has not addressed ECG abnormalities as potential predictors of mortality. The relationship between metabolic and electrolyte disturbances, ECG parameters, and the signs and symptoms of MAP are not well understood. As a result, our goal was to demonstrate the relationship between mortality and morbidity in MAP patients, as well as their ECG parameters, arterial blood gas results, symptoms, and late effects.

## 2. Materials and Methods

***Research Context***: Between 1 January 2017 and 31 December 2023, this retrospective research was conducted on patients admitted to the Emergency Medicine Clinic of the University of Health Sciences Bağcılar Training and Research Hospital in Istanbul, Turkey, as a result of MAP.

***Ethical Consideration***: On 22 March 2024, the University of Health Sciences Bagcilar Training and Research Hospital’s Non-Interventional Clinical Research Ethics Committee approved this study with Decision No. 2024/03/08/032. We obtain “informed consent” from hospital applications, stating that while their identity information will remain confidential, we can use their age, gender, and all laboratory and imaging data in the study, given the difficulty of reaching patients in retrospective studies. In addition, the hospital administration and the local ethics committee were assured that participants’ personal, contact information, and identity data would not be shared in any way. We strictly adhered to the Declaration of Helsinki for Research Projects at every stage of this study.

***Sample Size***: Investigating the frequency of methyl alcohol poisoning in the literature revealed a significant challenge in obtaining clear information. When we looked at all the references we used in our study, it was seen that the average of the selected cases was below 100 (minimum: 9–maximum: 383). As a result of the literature review, we were unable to find a publication suitable for our study, so we had to determine the sample size by conducting a pilot study. We performed a power analysis using the data from the pilot study and calculated the sample size required for our study. For this, G*Power [17] analysis was used, 95% confidence (1 − α), 95% test power (1 − β), and d = 0.5 effect size were determined according to the independent samples t test analysis. Between the study’s specified dates, our emergency department admitted a total of 5287 patients due to alcohol consumption. G*Power conducted an analysis among the cases diagnosed with MAP, excluding other alcohol causes, and found a population size of 5287 individuals, with an acceptable margin of error of 5% and a confidence interval of 95%. The analysis resulted in the inclusion of 227 patients in the study. In Turkey, there is a health system called ‘e-nabız’. By entering their ID numbers, patients can use this system to access information about their hospital examination in Turkey, their complaints, diagnosis, treatment, laboratory results, and imaging. Once the ethics committee accepts the application, hospital management and assigned healthcare personnel can view this system. This system allows for the monitoring of patient conditions. Data for all patients who applied between the specified dates were available. The study randomly included 227 cases with power analysis after excluding other alcohol-related poisonings and overdoses.

***Inclusion and Exclusion Criteria***: The study included cases diagnosed with eMAP in our Emergency Medicine Clinic between 1 January 2017 and 31 December 2023 that met the specific inclusion criteria. It was decided that a person had MAP if they met all of the following conditions: they had a high anion gap metabolic acidosis with no other known cause; their arterial pH was less than 7.3; their bicarbonate level was less than 20 mmol/L; and they had made or drunk alcohol in the last four days without measuring their methyl alcohol level. The diagnosis was based on the presence and absence of similar signs and symptoms in individuals who consumed the same MA [7]. The investigation included patients over the age of 18 who met the aforementioned criteria and had complete demographic, arterial blood gas, and electrocardiogram (ECG) data. The study excluded patients with pancreatitis, renal failure, chronic cardiac disease, cerebrovascular disease, and other substance use histories, as well as those who were younger than 18 years of age and lacked demographic data, arterial blood gas, and ECG data. The study excluded patients with pancreatitis and renal failure to assess the 6-month follow-up after treatment and determine if their complications were new.

***Electrocardiography***: We take a 12-lead ECG at the bedside with the Cardiofax ECG-9132K (Nihon Kohden, Tokyo, Japan) within the first 20 min of a patient’s admission to our emergency department as standard procedure. These ECG findings included atrial fibrillation, accelerated idioventricular rhythm, and right and left bundle branch block. All of these were new changes.

***QTc Calculation***: The QTc interval was recorded using the standard 12-lead ECG and uploaded to the automation system after MAP was defined. The Q interval, which is defined as the duration from the onset of the QRS complex to the conclusion of the T wave in the cardiac electrical cycle, was accurately evaluated due to the ECG device’s simultaneous recording of all leads. Two experienced clinicians measured the QT and RR intervals by observing five consecutive beats in lead DII. The Bazett formula was employed to rectify the QT interval for heart rate (QTc interval = QT/RR^1/2^) [18]. Abnormally prolonged QTc measurements were defined as ≥450 ms in men and ≥460 ms in women [19]. Nevertheless, in order to establish standardized data, three categories were established for both men and women: “short” QTc for individuals with a QTc of less than 349 ms, “normal” for those with a QTc of 350–449 ms, and “long” for those with a QTc of 450 ms or longer. In addition to the baseline ECG, patients who were discharged following MAP treatment underwent a standard 12-lead ECG six months following the MAP attack to assess the change in the QTc interval. For this ECG, patients followed up within 6 months after discharge were contacted for follow-up and called to the emergency department.

***Data Collection***: Our hospital’s registration system encompasses demographic, clinical, and laboratory data, as well as diagnoses, admission dates, and contact information. We analyzed the comorbid conditions of the patient population but found no condition that would influence the results. This situation was detected through hospital records, patient files, and the e-nabız system. We recorded the following patient data: age, gender, admission time, blood gas, serum lactate, hemogram, biochemistry, ECG, and mortality. The treatment of all cases was coordinated during their emergency admission. Six months after the MAP, we reviewed the patients’ hospital admissions. We kept records of the events that occurred during this period.

***Time***: It was defined as the approximate time between the patients starting to take MA and presenting to the emergency department.

***Laboratory Finding***: Venous blood gas, lactate, blood sugar, hemogram, and biochemical blood samples of the patients were taken during admission to the emergency department. Blood samples from all patients were taken within the first 20 min to establish standards. Venous blood gas levels of the patients were analyzed using the Acobas^®^ b221 Blood Gas System (Roche, Basel, Switzerland). The laboratory received the blood immediately after collection and analyzed the results within 5–10 min.

## 3. Statistical Analysis

The SPSS 20.0 software application (SPSS Inc., Chicago, IL, USA) was used to analyze the data obtained from this study. We implemented a one-sample Kolmogorov–Smirnov test to determine whether the variables derived from a normal distribution. We implemented the Kruskal–Wallis H-test and Mann–Whitney U test to evaluate the disparities between groups, given that the variables did not derive from a normal distribution. Chi-square analysis was implemented to investigate the relationships between nominal variables. In order to ascertain the correlation between groups, Spearman’s Rho correlation analysis was implemented. Additionally, we used the statistically significant variables from the univariate linear regression analysis in the multivariate linear regression risk model, applying the forward stepwise correction method to identify the independent prognostic factor for the development of MAP and mortality. We employed receiver operating characteristic (ROC) curve analysis to determine the sensitivity and specificity of QTc (ms) and pH values in relation to mortality. The results were interpreted as statistically significant when *p*-values were less than 0.05.

## 4. Results

The mean age of 227 cases included in the study was 43.23 ± 11.11 years, 8 (3.5%) were female, and the age distribution was 19–68 years. The age difference between QTc groups was not found to be significant (*p* = 0.792). Among the MAP and QTc groups, the QTc of the short group was measured at 343.22 ± 7.38 ms, and the distance of the long group was measured at 491.48 ± 14.8 ms (*p* < 0.001). The mean admission time of all patients (n = 227) was 19.1 ± 4.61 h (*p* < 0.001), blood sugar was 130.7 ± 32.09 mg/dL (*p* < 0.001), and osmolarity was 288.1 ± 21.84 mmol/kg (*p* = 0.533). In venous blood gases, mean pH of the cases (n = 227) was 7.14 ± 0.2 (*p* < 0.001), pO_2_ was 91.14 ± 2.16 mmol/L (*p* = 0.002), potassium was 5 ± 0.79 mmol/L, bicarbonate was 17.17 ± 4.86 mmol/L, based deficit was −6.21 ± 3.18 mmol/L, anion gap was 19.36 ± 7.31 mmol/L, and lactate was 4.82 ± 2.45 mmol/L (*p* < 0.001). Among the complaints of the patients, nausea/vomiting was detected in 63 (27.8%) patients, vision problems in 55 (24.2%) patients, change in consciousness in 57 (25.1%) patients, and abdominal pain in 48 (21.1%) patients. Normal sinus rhythm in 200 (88.1%) of the cases, sinus tachycardia in 31 (13.7%), bradycardia in 8 (3.5%), atrial fibrillation in 5 (2.2%), and accelerated idioventricular dysfunction in 3 (1.3%). rhythm, left bundle branch block in 11 (4.8%), and right bundle branch block in 22 (9.7%). According to these findings, gender, time elapsed until emergency admission, blood sugar, pH value, pO_2_, potassium, bicarbonate, base deficit, anion gap, and lactate levels were significantly higher in the QTc longer than 450 ms group (Table 1).

The mean age of patients with mortality (n = 49) was 44.84 ± 10.54 years, 47 (95.9) of whom were male. No relationship was found between age, gender, and mortality. However, QTc interval was 514.08 ± 5.45 ms, blood sugar was 156.69 ± 39.89 mg/dL, pH was 6.82 ± 0.14 mmol/L, bicarbonate was 12.60 ± 3.43 mmol/L, base deficit was −6.21 ± 3.18 mmol/L, anion gap was 23.49 ± 7.37 mmol/L, and lactate was 7.96 ± 1.93 mmol/L in the deceased patients. Significant results were found in the group with mortality compared to the survivors. All of the cases with mortality were intra-patient deaths. When the patient files, e-nabız, and hospital records of the cases were examined, no bradycardia, atrial fibrillation, accelerated idioventricular rhythm, or right and left bundle branch block were detected in the history of the patients. However, new ECG findings were seen in the ECG of the deceased cases. It was determined that the right bundle branch block accompanied this association most frequently (Table 2).

In the correlation analysis performed with variables of QTc groups, there was a strong positive correlation with QTc distance (r = 0.931) and time to admission (r = 0.507) (*p* < 0.001). Positive weak-moderate analysis was detected with blood sugar (r = 0.284), potassium (r = 0.411), anion gap (r = 0.182), and lactate (r = 0.364) (*p* < 0.001).Whereas there was a negative moderate to strong correlation with pH (r = −0.580), there was a negative weak to moderate correlation with pO_2_ (r = −0.211), bicarbonate (r = −0.376), and base deficit (r = −0.333) (*p* < 0.001). In the correlation analysis of these variables with mortality, positive correlations were found with QTc distance (r = 0.559), time to admission (r = 0.700), potassium (r = 0.788), lactate (r = 0.695), and negative strong correlations were found with pH (r = −0.874) and bicarbonate (r = −0.510). In addition, a weak to moderate positive correlation was found with blood sugar (r = 0.443) and anion gap (r = 0.302) levels. A weak to moderate negative correlation was found only with a base deficit (r = −0.307) (Table 3).

In the univariate and multivariate regression analysis performed with variables for mortality and QTc groups, blood sugar, QTc, pH, bicarbonate, base deficit, anion gap, and lactate were found to be significant in both groups in univariate analysis. In the multivariate analysis of the QTc group, only QTc was found to be a diagnostic value (*p* < 0.001). In the multivariate analysis performed for mortality, it was determined that blood sugar, QTc, pH, base deficit, and lactate could be predictive values in methyl alcohol poisoning (Table 4).

**Figure 1:**
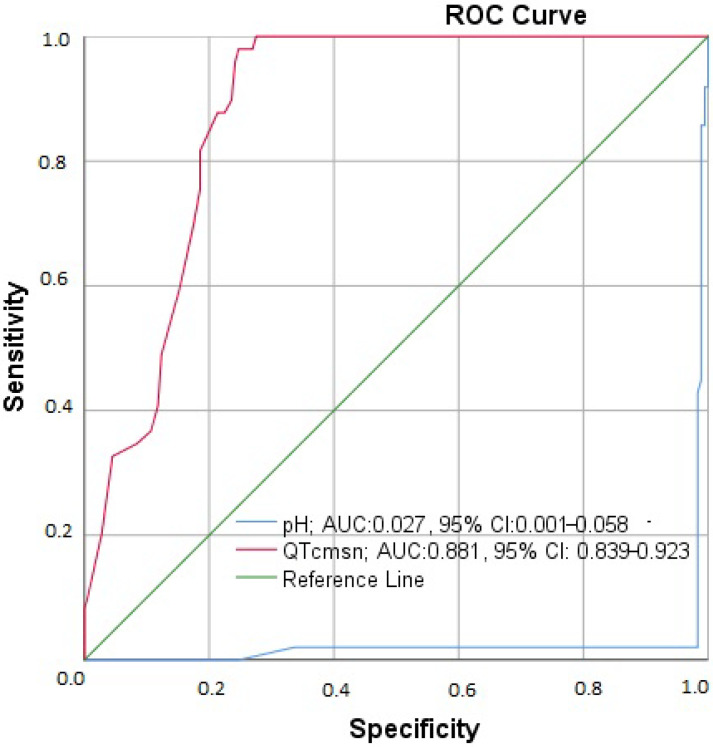
Receiver Operating Characteristic (ROC) Curve analysis graph showing QTc interval and pH levels for mortality.

In the treatment planning of the cases, fluid (100%) and 10% ethanol (100%) treatment was started in all cases. Additionally, fomepizole was administered to 56 (24.7%) cases, and hemodialysis was administered to 89 (39.2%). Patients were followed up for 6 months after they recovered from acute exposure and were discharged. No complications developed in 124 (54.6%) of these cases. Neurological deficits were observed in 4 (1.8) of the patients during their follow-up. In the imaging performed, areas of bleeding and necrosis were detected in two cases. Additionally, acute coronary syndrome and severe heart failure developed in 15 (6.6%) cases. Renal failure and pancreatitis occurred in six patients during follow-up. At the time of presentation to the emergency department, 55 (24.4%) patients had visual complaints. Permanent blindness was observed in 7 of them in the acute phase and 23 (10.1%) in the late phase after treatment (Table 5). An analysis of mortality regarding QTc and pH is given in Figure 1.

## 5. Discussion

Numerous recent studies have explored the acute effects of methyl alcohol poisoning, which has been the subject of numerous recent studies. However, we have not come across any studies in the literature regarding the late complications of MAP, such as permanent blindness, neurological deficit, cardiac deficit, renal failure, and pancreatitis, after treatment in the acute phase. This prompted us to explore the long-term consequences of MAP for individuals. We showed that people who go to the emergency room 23 h after taking MA are at a high risk of developing late neurological, cardiac, permanent blindness, diabetes, renal failure, and pancreatitis. This risk is the same for people of all ages. In addition, we discovered that the most significant predictive value for in-hospital mortality may be a pH value below 6.93 and a QTc distance exceeding 514 ms. Consequently, MA is the first study to assess the combined effects of in-hospital mortality and late-term effects.

Our cases averaged 43 years old, and 96.5% of them were male. 328 of 356 patients (89.9%) were male in similar studies conducted by Nikoo et al. [6], with a mean age of 32.76 ± 10.61 years. 97.2% of the 109 cases studied by Abdelwahab et al. [20] were male, with an average age of 37.9 years. We believe that these findings may be associated with the fact that males are more likely to consume alcohol in general and produce alcohol at home. Despite reports of methyl alcohol exposure through various routes, we most frequently used the oral route [21]. MA is toxic at a concentration of 8–10 mL, and a single dose of approximately 25–30 mL can result in permanent blindness. Ingestion of 1 mL/kg, or 100 mL, is fatal. MA is rapidly absorbed from the gastric mucosa and reaches its maximal plasma concentration within 30–60 min when administered orally [22]. The enzyme alcohol dehydrogenase converts MA to formaldehyde, while the enzyme aldehyde dehydrogenase converts it to formic acid. Numerous tissues are susceptible to formic acid’s toxic effects. Cellular dysfunction and end-organ injury are the consequences of cytochrome-c oxidase inhibition in the electron transport chain. This results in an increase in lactate and metabolic acidosis. Subsequently, three distinct clinical phases transpire. The first stage is characterized by nonspecific gastrointestinal complaints, while the second stage is characterized by a brief latent period that is comparable to the symptoms that occur following classical alcohol consumption. This period may extend for a maximum of 48 h. Blurred or double vision, photophobia, altered consciousness, and early or late blindness may accompany end-stage severe metabolic acidosis [23]. We determined that the most common complaints from our patients included nausea, vomiting, abdominal pain, change of consciousness, and impaired or lost vision.

Our patients, who were discharged with complete recovery following acute exposure, were monitored for six months. In patients who did not have any comorbidities before MAP, late findings or complications developed as a result of the MA’s effects.

I. Fifty-five (24.1%) patients presented with visual complaints in the acute phase. Seven of these cases were blind upon admission to the emergency department and did not respond to treatment. We detected permanent vision loss in 23 (10.1%) cases who underwent follow-up within 6 months after discharge. The mean QTc interval of the patients with permanent blindness was 512 ms, pH was below 7.0, base deficit was −6.6 mmol/L, lactate was 3.76 mmol/L, bicarbonate was 17.3 mmol/L, and blood sugar was 124 mg/dL. In other words, in cases with permanent blindness, acidosis was more severe, parameters were worse, and the QTc interval was much longer than normal. Central scotoma and papilledema were present in all cases of blindness. Studies that employed MA blinding yielded comparable outcomes. In their study, Newman et al. [24] found that MAP damages the optic nerve and retinal epithelial cells, resulting in symmetrical or asymmetric impaired vision or blindness in severe poisoning. These findings were caused by formic acid and may not appear for 48–72 h. They asserted that the eye examination is characterized by central scotoma, erythema, pallor of the optic disc, and papilledema.

II. The investigation admitted 57 (25.1%) cases of altered consciousness. We observed neurological deficits in four of our discharged cases after treatment and during their six-month follow-up. We observed hemorrhage in the vicinity of the putamen and basal ganglia in two of these cases. The other two cases exhibited Parkinsonism-like characteristics. All of these cases were found to be associated with blindness. The inhibition of presynaptic gamma-aminobutyric acid (GABA) receptors, N-methyl-D-aspartic acid (NMDA) glutamate receptors, and an increase in GABA levels are the neurological findings of MA. Patients may experience headaches, central nervous system depression, coma, and seizures [25]. Computed tomography or magnetic resonance imaging can detect putaminal necrosis, intracranial hemorrhage, basal ganglion lesions, necrosis in the putamen, and aberrant appearances in the caudate nucleus [26]. A poor prognosis is associated with the presence of these lesions. Permanent sequelae may result from cerebral and eye lesions [3].

III. Despite the absence of any prior complaints, we identified cardiac complaints in 15 (6.6%) of our cases within six months of the onset of methyl alcohol toxicity. We diagnosed acute coronary syndrome (ST-elevation myocardial infarction in six, non-ST-elevation myocardial infarction in three), severe heart failure with a left ventricular ejection fraction of less than 35% in five, and myopericarditis in one patient in nine of them. Despite the absence of a condition in the hospital records of these cases, it is important to remember that MAP or coincidental factors could trigger it. Further research is required to address this. The literature review indicated that there were limited studies on the cardiovascular effects of MA. We have documented several ECG abnormalities, including sinus tachycardia, T wave alterations, and increased PR and QTc intervals [4]. It can also result in acute coronary syndrome and severe cardiac failure [27]. One case report showed that the right bundle branch block developed along with the left anterior fascicular block, paroxysmal atrial fibrillation, and an increase in the end-diastolic and end-systolic sizes of the left ventricle. They reported a 5.3% prevalence rate for myocardial infarction. We propose the following hypotheses concerning myocardial infarction: (a) Infarction is possible in patients who are relatively young and lack the traditional risk factors for atherosclerosis. (b) Severe acidosis, resulting from a reduction in fibrinogen levels, causes bleeding. In situ, thrombosis may occasionally develop in conjunction with ongoing disseminated intravascular coagulation and the potential for myocardial infarction. (c) The potential pathophysiological mechanism is endothelial dysfunction, a condition that is associated with diminished extracellular pH. However, this dysfunction would lead to vasodilation. A mechanism of myocardial infarction cannot explain the four (d) spasms, as acidosis induces vasodilation [14]. Nikoo et al. [6] conducted an additional investigation and found that the frequent occurrence of the U wave, elevation of the J point, and a QTc longer than 500 ms typically indicate the severity of the condition. 25.3% of the sample had a heart rate exceeding 100, nearly six times higher than bradycardia. Furthermore, the majority of prior investigations have documented QT prolongation [28,29]. In reality, our investigation yielded comparable outcomes. The automation system indicated that all of our cases’ previous electrocardiograms were in normal sinus rhythm. Normal sinuses persisted in 200 patients (88.1%) following methyl alcohol toxicity. Nevertheless, 31 (13.7%) patients experienced tachycardia, 8 (3.5%) individuals experienced bradycardia, 5 (2.2%) patients developed newly developed atrial fibrillation, 3 (1.3%) individuals experienced accelerated idioventricular rhythm, and 11 (4.8%) patients experienced a new onset of atrial fibrillation. We identified left bundle branch blocks and right bundle branch blocks in 22 cases (9.7%). Nevertheless, the control electrocardiograms obtained at discharge following treatment indicated that all had improved. The investigation by Nikoo et al. [6] was comparable, and the most noteworthy finding was that the QTc interval exceeded 512 milliseconds. In addition, the tachycardia/bradycardia ratio was approximately 3.9.

IV. Pancreatitis and renal failure occurred in six cases each during the six-month follow-up. We identified pseudocysts in two of the pancreatitis cases and detected bile sediment and stones in the other cases. The absence of pre-MA ultrasonography information made it impossible to definitively link MAP to the cases, but it was still considered a predisposing factor. The literature recognizes gallstone disease, smoking, and excessive alcohol consumption as risk factors for both acute and chronic pancreatitis. Nevertheless, the cause of approximately 20% of pancreatitis cases is idiopathic, and the exact cause is not yet known [30,31]. However, the incidence of acute pancreatitis following methyl alcohol consumption is not well understood, even though ethanol consumption is one of the primary causes of acute and chronic pancreatitis. Chronic pancreatitis accounts for approximately 8% of acute pancreatitis [32]. Histological changes in the liver tissue as a result of methyl alcohol, micro- and macrovesicular steatosis, central hepatocyte necrosis, intrahepatic biliary stasis, and hydropic degeneration may be the cause of pancreatitis [33]. After MA, acute kidney injury and pancreatitis have been documented [34,35]. Myoglobinuria may be the cause of an acute kidney injury resulting from MA. Elevated osmolality, concentration, and a low pH may also be associated with it [36]. Velvet et al. [37] observed hydropic alterations in the proximal tubule in kidney biopsies of patients who died from MAP.

V. Mortality: In-hospital fatalities comprised each of our cases. In these instances, the time to emergency department admission was greater than 25 h, the pH was less than 6.8, the blood sugar was greater than 156 mg/dL, the bicarbonate was less than 12.6 mmol/L, the base gap was less than −8 mmol/L, the angon gap was greater than 23.5 mmol/L, the lactate was greater than 7.9 mmol/L, and the QTc interval was greater than 514 ms. The most significant findings were QTc (HR: 0.509, 95%CI: 0.005–0.006) and pH (HR: 0.764, 95%CI: −1.929–−1.667), despite the fact that all of these factors were predictive of mortality. Investigations have indicated that mortality rates are elevated in the absence of early intervention for MAP patients. A mortality rate of 38.9% was determined in a single-case series. Independent predictors of mortality included a high lactate level, a low Glasgow coma scale score, and a delay in hospital admission. When the lactate level exceeded 5.75 mmol/L, mortality rates increased rapidly [34]. The MAP mortality rate in Iran has been reported to fluctuate between 7% and 51% [38]. This discrepancy in MAP mortality rates among various studies may be attributable to variations in geographic region, race, pattern of MA consumption, timely diagnosis of intoxication, standard supportive care, correction of metabolic acidosis, and other underlying factors. A pH value of ≤6.9 was significantly associated with mortality, as reported by Tabatabaie et al. [39] and Navabi et al. [40]. It was also determined that a poor prognosis was predicted by coma, seizure, and severe metabolic acidosis at the time of admission. In the intensive care unit, Smuszkiewicz et al. [41] found that both lactate and base deficits have prognostic value in predicting mortality.

All of our cases were given hydration and ethanol treatment when they presented to the emergency department. Fomeprizole was also administered to 56 patients who were intubated and whose general condition was poor. Hemodialysis was also performed in 89 cases. Bicarbonate treatment was added to patients with pH values <7.3. In the literature, the basic principle of MAP treatment is rapid diagnosis and resuscitation. Providing cardiopulmonary support, preventing the transformation of toxic metabolites, correcting existing metabolic acidosis, and removing toxic metabolites from the body are all very important [42]. Methyl alcohol often causes hypotension through vasodilation and vomiting, and many patients require hydration with intravenous crystalloid. The most important part of initiating MAP treatment for known or suspected cases is blocking alcohol dehydrogenase, which prevents the formation of toxic metabolites. To achieve this, administer ethyl alcohol intravenously (IV) as 10% ethanol, starting with a loading dose of 10 mL/kg and gradually increasing to 1 mL/kg/h, resulting in a serum concentration of approximately 150 mg/dL. In addition, fomepizole is a competitive antagonist for the enzyme alcohol dehydrogenase, with a 15 mg/kg IV loading dose followed by 10 mg/kg IV every 12 h for up to 4 doses [2]. Hemodialysis is the best method to rapidly eliminate both toxic acid metabolites and parent alcohols, and it plays a fundamental role in the treatment of severely poisoned patients. Additionally, patients can receive sodium bicarbonate, folic acid, and corticosteroids for ocular findings [43].

## 6. Study Limitations

The most important limitation is that the report is retrospective and may introduce monocentric bias. Furthermore, we cannot establish a direct causal relationship between pH and ECG changes due to the challenge of distinguishing their effects from concurrent biochemical deviations or drug interactions. Additionally, it was desirable to have baseline and follow-up ECGs. Finally, the observed changes may necessitate a larger prospective observational study.

## 7. Conclusions

This study evaluated the effects of methyl alcohol on human ECG and late findings. We detected no specific ECG findings or patterns, despite the fact that methyl alcohol poisoning causes various ECG changes that may depend on the severity of acidosis. However, the study demonstrated that QTc prolongation and low pH values can serve as predictive values for both mortality and late findings. It will also guide future studies, as it is the first study to evaluate late findings.

## Figures and Tables

**Table 1:** Basic characteristics of methyl alcohol poisoning according to QTc groups and analysis of laboratory results.

Methyl Alcohol Poisoning						
		All Patient n: 227 Mean ± SD	QTc Groups			*p* Value
			Short (Less than 349 ms) n: 8 Mean ± SD	Normal (350–449 ms) n: 115 Mean ± SD	Long (Longer than 450 ms) n: 104 Mean ± SD	
Age, year		43.23 ± 11.11	45.44 ± 12.22	42.88 ± 11.52	43.79 ± 9.99	0.792
Gender	Female n (%)	8 (3.5)	0	1 (0.4)	7 (3.1)	0.001 *
	Male n (%)	219 (96.5)	9 (4)	154 (68.3)	56 (27.8)	
QTc, ms		442.77 ± 60.07	339.5 ± 5.42	388.17 ± 26.6	511.1 ± 16.51	**<0.001**
Time, hour		19.10 ± 4.61	20.33 ± 4.61	17.22 ± 3.00	23.57 ± 4.79	**<0.001**
Blood sugar, mg/dL		130.70 ± 32.09	127.89 ± 22.79	124.22 ± 25.43	147.05 ± 41.28	**<0.001**
Osmolarity, mmol/kg		288.10 ± 21.84	293.18 ± 9.23	287.35 ± 25.23	289.21 ± 11.84	0.533
Venous Blood Gas	pH	7.14 ± 0.20	7.13 ± 0.20	7.23 ± 0.10	6.93 ± 0.22	**<0.001**
	pCO_2_, mmHg	36.96 ± 4.17	36.00 ± 4.82	36.79 ± 4.11	37.51 ± 4.21	0.360
	pO_2_, mmHg	91.14 ± 2.16	91.20 ± 1.45	91.48 ± 2.09	90.32 ± 2.20	**0.002**
	Potassium, mmol/L	5.00 ± 0.79	5.45 ± 0.92	4.70 ± 0.56	5.66 ± 0.84	**<0.001**
	Calcium, mmol/L	1.21 ± 0.25	1.06 ± 0.27	1.21 ± 0.22	1.23 ± 0.32	0.591
	Sodium, mmol/L	137.24 ± 3.50	137.92 ± 3.46	137.22 ± 3.64	137.19 ± 3.20	0.817
	Chlorine, mmol/L	100.46 ± 5.75	100.03 ± 5.09	100.19 ± 5.56	101.21 ± 6.31	0.446
	Bicarbonate, mmol/L	17.17 ± 4.86	17.32 ± 5.20	18.51 ± 4.37	13.83 ± 4.42	**<0.001**
	Base Deficit, mmol/L	−6.21 ± 3.18	−4.82 ± 4.02	−5.58 ± 3.24	−7.95 ± 2.12	**<0.001**
	Anion Gap, mmol/L	19.36 ± 7.31	21.00 ± 7.58	18.18 ± 6.74	22.05 ± 7.96	**0.002**
	Lactate, mmol/L	4.82 ± 2.45	5.51 ± 3.25	4.08 ± 1.79	6.56 ± 2.84	**<0.001**
Application Complaint		Nausea/Vomiting, n (%)				63 (27.8)
		Vision Loss, n (%)				55 (24.2)
		Altered Consciousness, n (%)				57 (25.1)
		Stomach Ache, n (%)				48 (21.1)
Electrocardiography		Normal Sinus Rhythm, n (%)				200 (88.1)
		Tachycardia, n (%)				31 (13.7)
		Bradycardia, n (%)				8 (3.5)
		Atrial Fibrillation, n (%)				5 (2.2)
		Accelerated Idioventricular Rhythm, n (%)				3 (1.3)
		Left bundle branch block, n (%)				11 (4.8)
		Right bundle branch block, n (%)				22 (9.7)

SD: Standard Deviation, QTc: Corrected QT interval, pH: Power of Hydrogen, pCO_2_: Partial Carbon Dioxide Pressure, pO_2_: Partial Oxygen Pressure, *p*: Statistical significance (<0.05), * Chi-square test was used for gender, while Kruskal–Wallis H-Test was used for other variables.

**Table 2:** Basal and laboratory findings of methyl alcohol poisoning according to mortality of patients.

		Mortality		
		No n: 148 Mean ± SD	Yes n: 49 Mean ± SD	*p* Value
Age, year		42.80 ± 11.26	44.84 ± 10.54	0.212
Gender	Female	6 (3.4)	2 (4.1)	0.684 *
	Male	172 (96.6)	47 (95.9)	
QTc, ms		423.15 ± 62.77	514.08 ± 5.45	**<0.001**
Blood sugar, mg/dL		123.55 ± 25.40	156.69 ± 39.89	**<0.001**
pH		7.23 ± 0.10	6.82 ± 0.14	**<0.001**
Bicarbonate, mmol/L		18.42 ± 4.43	12.60 ± 3.43	**<0.001**
Base Deficit, mmol/L		−5.70 ± 3.33	−6.21 ± 3.18	**<0.001**
Anion Gap, mmol/L		18.23 ± 6.89	23.49 ± 7.37	**<0.001**
Lactate, mmol/L		3.96 ± 1.79	7.96 ± 1.93	**<0.001**
Electrocardiography		Bradycardia, n (%)		5 (10.2)
		Atrial Fibrillation, n (%)		4 (8.2)
		Accelerated Idioventricular Rhythm, n (%)		3 (6.1)
		Left bundle branch block, n (%)		6 (12.2)
		Right bundle branch block, n (%)		12 (24.5)

SD: Standard Deviation, QTc: Corrected QT interval, pH: Power of Hydrogen, *p*: Statistical significance (<0.05), * Chi-square test was used for gender, while the Mann–Whitney U test was used for other variables.

**Table 3:** Correlation analysis of methyl alcohol poisoning QTC groups and mortality with variables.

Methyl Alcohol Poisoning				
	QTc Groups		Mortality	
	r	*p* Value	r	*p* Value
QTc, ms	**0.931**	**<0.001**	**0.559**	**<0.001**
Time, hour	**0.507**	**<0.001**	**0.700**	**<0.001**
Blood sugar, mg/dL	**0.284**	**<0.001**	**0.443**	**<0.001**
pH	**−0.580**	**<0.001**	**−0.874**	**<0.001**
pO_2_, mmHg	**−0.211**	**0.001**	−0.115	0.084
Potassium, mmol/L	**0.411**	**<0.001**	**0.788**	**<0.001**
Bicarbonate, mmol/L	**−0.376**	**<0.001**	**−0.510**	**<0.001**
Base Deficit, mmol/L	**−0.333**	**<0.001**	**−0.307**	**<0.001**
Anion Gap, mmol/L	**0.182**	**0.006**	**0.302**	**<0.001**
Lactate, mmol/L	**0.364**	**<0.001**	**0.695**	**<0.001**

r: correlation coefficient, QTc: Corrected QT interval, pH: Power of Hydrogen, pO_2_: Partial Oxygen Pressure, *p*: Statistical significance (<0.05).

**Table 4:** Regression analysis of methyl alcohol poisoning with QTc groups and mortality with variables.

Methyl Alcohol Poisoning							
		Univariate			Multivariate		
		HR	95% Cl	*p*	HR	95% Cl	*p*
MortalityQTc Groups	Blood sugar, mg/dL	**0.081**	**0.003–0.007**	**<0.001**		−0.001–0.001	0.536
	QTc, ms	**0.867**	**0.007–0.008**	**<0.001**		**0.007–0.008**	**<0.001**
	pH	**0.336**	**−1.754–−1.207**	**<0.001**		−0.326–0.192	0.611
	Bicarbonate, mmol/L	**0.141**	**−0.052–−0.027**	**<0.001**		−0.010–0.008	0.844
	Base Deficit, mmol/L	**0.111**	**−0.073–−0.034**	**<0.001**		−0.014–0.010	0.710
	Anion Gap, mmol/L	**0.033**	**0.004–0.022**	**0.006**		−0.006- 0.004	0.742
	Lactate, mmol/L	**0.133**	**0.050–0.101**	**<0.001**		−0.023–0.012	0.538
	Blood sugar, mg/dL	**0.196**	**0.004–0.007**	**<0.001**		**0.001–0.002**	**0.001**
	QTc, ms	**0.313**	**0.003–0.004**	**<0.001**		**0.001–0.002**	**0.013**
	pH	**0.764**	**−1.929–−1.667**	**<0.001**		**−1.302–−0.965**	**<0.001**
	Bicarbonate, mmol/L	**0.261**	**−0.053–−0.034**	**<0.001**		−0.010–0.002	0.217
	Base Deficit, mmol/L	**0.094**	**−0.056–−0.024**	**<0.001**		**0.002–0.018**	**0.012**
	Anion Gap, mmol/L	**0.091**	**0.010–0.024**	**<0.001**		−0.005–0.002	0.329
	Lactate, mmol/L	**0.482**	**0.101–0.133**	**<0.001**		**0.027–0.049**	**<0.001**

HR: Hazard Ratio, 95% CI: Confidence Interval, QTc: Corrected QT interval, pH: Power of Hydrogen, *p*: Statistical significance (<0.05).

**Table 5:** Treatment used in methyl alcohol poisoning and 6-month follow-up results after discharge.

Methyl Alcohol Poisoning					
**Treatment Application**			**Follow-Up**		
Treatment n(%)	Liquid	227 (100)	Six-month follow-up n(%)	No	124 (54.6)
	Ethanol	227 (100)		Neurological deficit	4 (1.8)
	Fomepizole	56 (24.7)		Cardiac deficit	15 (6.6)
	Hemodialysis	89 (39.2)		Permanent blindness	23 (10.1)
	Intubation	56 (24.7)		Kidney failure	6 (2.6)
				Pancreatitis	6 (2.6)
				Mortality (In-hospital deaths)	49 (21.6)
**Receiver Operating Characteristic (ROC) Curve analysis with variables for mortality**					
Mortality	**Sensitivity (%)**	**Specificity (%)**	**AUC**	**95% CI**	***p*-value**
QTc, ms	99.4	98.0	0.881	0.839–0.929	**<0.001**
pH	99.6	99.2	0.027	0.000–0.058	**<0.001**

AUC: Area Under the Curve, 95% CI: Confidence Interval, *p*: Statistical significance (<0.05).

## Data Availability

All data is available on request without restriction.

## References

[1] Ocak T., Kalafat U.M., Basturk M. (2016). Alcohol Poisonings. Turk. Clin. J. Emerg. Med-Spec. Top..

[2] Ott J., Gronemann V., Pontzen F., Fiedler E., Grossmann G., Kersebohm D.B., Weiss G., Witte C. (2012). Methanol. Ullmann’s Encyclopedia of Industrial Chemistry.

[3] Taşkın Ö., Akpınar A.A., Dişel N.R. (2022). Methyl Alcohol Intoxications. Anatol. J. Emerg. Med..

[4] Jaff Z., McIntyre W.F., Yazdan-Ashoori P., Baranchuk A. (2014). Impact of methanol intoxication on the human electrocardiogram. Cardiol. J..

[5] Theobald J., Lim C. (2019). Folate as an adjuvant therapy in methanol poisoning. Nutr. Clin. Pract..

[6] Nikoo M.H., Arjangzadeh A., Pakfetrat M., Boogar S.S., Mohammadkarimi V., Ostovan V.R., Khodamoradi Z., Roozbeh J., Khalili M., Shirazi F.K.H. (2020). Electrocardiographic findings of methanol toxicity: A cross-sectional study of 356 cases in Iran. BMC Cardiovasc. Disord..

[7] Kurtas O., Imre K.Y., Ozer E., Can M., Birincioglu İ., Butun C., Kırcı G.S., Yıldırım A., Kıyak S., Yılmaz R. (2017). The evaluation of deaths due to methyl alcohol intoxication. Biomed. Res..

[8] Liberski S., Kaluzny B.J., Kocięcki J. (2022). Methanol-induced optic neuropathy: A still-present problem. Arch. Toxicol..

[9] Zakharov S., Kotikova K., Nurieva O., Hlusicka J., Kacer P., Urban P., Vaneckova M., Seidl Z., Diblik P., Kuthan P. (2017). Leukotriene-mediated neuroinflammation, toxic brain damage, and neurodegeneration in acute methanol poisoning. Clin. Toxicol..

[10] Hlusicka J., Loster T., Lischkova L., Vaneckova M., Seidl Z., Diblik P., Kuthan P., Urban P., Navratil T., Kacer P. (2018). Role of activation of lipid peroxidation in the mechanisms of acute methanol poisoning. Clin. Toxicol..

[11] Schep L.J., Slaughter R.J., Vale J.A., Beasley D.M. (2009). A seaman with blindness and confusion. BMJ.

[12] Hovda K.E., Hunderi O.H., Rudberg N., Froyshov S., Jacobsen D. (2004). Anion and osmolal gaps in the diagnosis of methanol poisoning: Clinical study in 28 patients. Intensive Care Med..

[13] Sanaei-Zadeh H., Emamhadi M., Farajidana H., Zamani N., Amirfarhangi A. (2013). Electrocardiographic manifestations in acute methanol poisoning cannot predict mortality. Arh. Hig. Rada Toksikol..

[14] Nikoo M.H., Estedal A., Pakfetrat M., Abtahi F., Heydari S.T. (2022). Mortality-related electrocardiogram indices in methanol toxicity. World J. Emerg. Med..

[15] Ceasovschih A., Șorodoc V., Covantsev S., Balta A., Uzokov J., Kaiser S.E., Almaghraby A., Lionte C., Stătescu C., Sascău R.A. (2024). Electrocardiogram Features in Non-Cardiac Diseases: From Mechanisms to Practical Aspects. J. Multidiscip. Healthc..

[16] Dibajnia P., Sivilotti M.L., Juurlink D., Shurrab M. (2020). ST-elevation in ethylene glycol toxicity mimicking myocardial infarction. J. Electrocardiol..

[17] Sut N. (2011). Sample size determination and power analysis in clinical trials. RAED J..

[18] Mirvis M.D., Goldberger L.A. (2006). Electrocardiografía. En: Braunwald, E. Tratado deCardiología.

[19] Rautaharju P.M., Surawicz B., Gettes L.S., Bailey J.J., Childers R., Deal B.J., Gorgels A., Hancock E.W., Josephson M., Kligfield P. (2009). American Heart Association Electrocardiography and Arrhythmias Committee Council on Clinical Cardiology; American College of Cardiology Foundation; Heart Rhythm Society. AHA/ACCF/HRS recommendations for the standardization and interpretation of the electrocardiogram: Part IV. The ST segment, T and U waves, and the QT interval: A scientific statement from the American Heart Association Electrocardiography and Arrhythmias Committee, Council on Clinical Cardiology; the American College of Cardiology Foundation; and the Heart Rhythm Society. Endorsed by the International Society for Computerized Electrocardiology. Circulation.

[20] Abdelwahab H.M., Nafea O.E., Elsherif R., Gharib A.F., Alrehaili A.A., Abdelhamid W.G. (2022). Neutrophil-to-lymphocyte ratio versus platelet-to-lymphocyte ratio in predicting clinical outcomes in acute methanol poisoning. Hum. Exp. Toxicol..

[21] Gómez Perera S., Rodríguez Talavera I., Tapia Quijada H.E., Guerrero-Mártir M., Díaz de Aguilar Osona M., Falcón Roca R. (2020). Secondary visual loss due to inhalation and cutaneous poisoning by methanol and toluene. Presentation of a clinical case. Arch. Soc. Esp. Oftalmol. (Engl. Ed.).

[22] Pohanka M. (2016). Toxicology and the biological role of methanol and ethanol: Current view. Biomed. Pap. Med. Fac. Univ. Palacky Olomouc Czechoslov..

[23] Paasma R., Hovda K.E., Hassanian-Moghaddam H., Brahmi N., Afshari R., Sandvik L., Jacobsen D. (2012). Risk factors related to poor outcome after methanol poisoning and the relation between outcome and antidotes—A multicenter study. Clin. Toxicol..

[24] Newman N., Biousse V. (2014). Diagnostic approach to vision loss. CONTINUUM Lifelong Learn. Neurol..

[25] Symington L., Jackson L., Klaassen B. (2005). Toxic alcohol but not intoxicated—A case report. Scott. Med. J..

[26] Jain N., Himanshu D., Verma S.P., Parihar A. (2013). Methanol poisoning: Characteristic MRI findings. Ann. Saudi Med..

[27] Poloková K., Hlinomaz O., Panovsky R. (2014). Acute anterior myocardial infarction caused by thrombotic occlusion of LAD in a patient with acute methanol intoxication. Interv. Akutni Kardiol..

[28] Ahmed F., Khan N.U., Ali N., Feroze A. (2017). Methanol poisoning: 27 years experience at a tertiary care hospital. J. Pak. Med. Assoc..

[29] Zakharov S., Pelclova D., Urban P., Navratil T., Diblik P., Kuthan P., Hubacek J.A., Miovsky M., Klempir J., Vaneckova M. (2014). *Czech mass methanol outbreak* 2012, epidemiology, challenges and clinical features. Clin. Toxicol..

[30] Yadav D., Lowenfels A.B. (2013). The epidemiology of pancreatitis and pancreatic cancer. Gastroenterology.

[31] Pang Y., Kartsonaki C., Turnbull I., Guo Y., Yang L., Bian Z., Chen Y., Millwood I.Y., Bragg F., Gong W. (2018). Metabolic and lifestyle risk factors for acute pancreatitis in Chinese adults: A prospective cohort study of 0.5 million people. PLoS Med..

[32] Petrov M.S., Yadav D. (2019). Global epidemiology and holistic prevention of pancreatitis. Nat. Rev. Gastroenterol. Hepatol..

[33] Akhgari M., Panahianpour M.H., Bazmi E., Etemadi-Aleagha A., Mahdavi A., Nazari S.H. (2013). Fatal methanol poisoning: Features of liver histopathology. Toxicol. Ind. Health.

[34] Hlusicka J., Mana J., Vaneckova M., Kotikova K., Diblik P., Urban P., Navratil T., Marechal B., Kober T., Zakharov S. (2020). MRI-based brain volumetry and retinal optical coherence tomography as the biomarkers of outcome in acute methanol poisoning. Neurotoxicology.

[35] Chang S.T., Wang Y.T., Hou Y.C., Wang I.K., Hong H.H., Weng C.H., Huang W.H., Hsu C.W., Yen T.H. (2019). Acute kidney injury and the risk of mortality in patients with methanol intoxication. BMC Nephrol..

[36] Nekoukar Z., Zakariaei Z., Taghizadeh F., Musavi F., Banimostafavi E.S., Sharifpour A., Ebrahim Ghuchi N., Fakhar M., Tabaripour R., Safanavaei S. (2021). Methanol poisoning as a new world challenge: A review. Ann. Med. Surg..

[37] Verhelst D., Moulin P., Haufroid V., Wittebole X., Jadoul M., Hantson P. (2004). Acute renal injury following methanol poisoning: Analysis of a case series. Int. J. Toxicol..

[38] Lee C.Y., Chang E.K., Lin J.L., Weng C.H., Lee S.Y., Juan K.C., Yang H.Y., Lin C., Lee S.H., Wang I.K. (2014). Risk factors for mortality in Asian Taiwanese patients with methanol poisoning. Ther. Clin. Risk Manag..

[39] Rafiei Tabatabaiei M.R., Yazdani S., Qorbani M., Shojaei Arani L., Faraji Dana H. (2022). Evaluation of Electrocardiogram Changes in Patients with Methanol Poisoning. Asia Pac. J. Med. Toxicol..

[40] Navabi S.J., Eivazi M., Beiranvand B. (2018). An epidemiological study of patients with methanol poisoning and the factors affecting the prognosis of patients in Imam Khomeini Hospital Kermanshah 2010–2015. Sci. J. Forensic Med..

[41] Smuszkiewicz P., Jawień N., Szrama J., Lubarska M., Kusza K., Guzik P. (2022). Admission lactate concentration, base excess, and alactic base excess predict the 28-day inward mortality in Shock patients. J. Clin. Med..

[42] Akyol S.A.J., Çete Y., Denizbaşı A., Çevik A.A., Oktay C., Atilla R. (2013). Alcohols. Tintinalli Emergency Medicine: A Comprehensive Study Guide.

[43] Sivilotti M.L.A. (2024). Methanol and Ethylene Glycol Poisoning: Pharmacology, Clinical Manifestations, and Diagnosis. Section Ed.: Burns, M.M., Hendrickson, R.G.; Deputy Ed.: Ganetsky, M. https://medilib.ir/uptodate/show/336.

